# Far‐Red Interlayer Excitons of Perovskite/Quantum‐Dot Heterostructures

**DOI:** 10.1002/advs.202207653

**Published:** 2023-03-20

**Authors:** Taek Joon Kim, Sang‐hun Lee, Eunji Lee, Changwon Seo, Jeongyong Kim, Jinsoo Joo

**Affiliations:** ^1^ Department of Physics Korea University Seoul 02841 Republic of Korea; ^2^ Department of Energy Science Sungkyunkwan University Suwon 16419 Republic of Korea; ^3^ Department of Physics and Energy Harvest‐Storage Research Center University of Ulsan Ulsan 44610 Republic of Korea

**Keywords:** charge transfer, far‐red, interlayer exciton, optoelectronics, perovskite, quantum dots

## Abstract

Interlayer excitons (IXs) at the interface of heterostructures (HSs) with a staggered band alignment are fascinating quantum quasi‐particles with light‐emitting and long‐lifetime characteristics. In this study, the energy band alignments (EBAs) of the HS of MAPbI_3_ perovskite thin sheets with CdSe‐ZnS core–shell quantum dot (QD) layers are modulated by using different diameters of the QDs. Far‐red IX emission is observed at 1.42 eV from the HS of MAPbI_3_/CdSe‐ZnS‐QD (*λ*_em_ = 645 nm) with type‐II EBA owing to charge transfer. The lifetime of the far‐red IXs is estimated to be 5.68 µs, which is considerably longer than that (0.715 ns) of the intralayer excitons from CdSe‐ZnS‐QD. With increasing incident excitation power, the PL peak and its intensity of IXs are blue‐shifted and linearly increased, respectively, indicating a strong dipole alignment of far‐red IXs at the heterojunction. Back focal plane imaging suggests that the directions of dipole moments of the IXs are relatively out‐of‐plane compared to those of the intralayer excitons (MAPbI_3_ and CdSe‐ZnS‐QD). Notably, the abnormal behavior of the optical characteristics is observed near the phase transition temperature (90 K) of MAPbI_3_. MAPbI_3_/CdSe‐ZnS‐QD HS photodetectors show the increase in photocurrent and detectivity compared to MAPbI_3_ at IX excitation.

## Graphical abstract

Heterostructures (HSs) of MAPbI_3_ perovskite with CdSe‐ZnS QDs are fabricated to study interlayer excitons (IXs). Far‐red IX emission is observed at 1.42 eV from the HS of MAPbI_3_/CdSe‐ZnS‐QD owing to charge transfer, and the lifetime of IXs is considerably long as 5.68 µs. MAPbI_3_/CdSe‐ZnS‐QD HS photodetectors show the increase in photocurrent and detectivity compared to MAPbI_3_ at IX excitation.

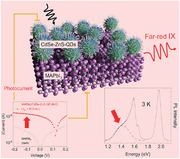

## 1 Introduction

Heterostructures (HSs) using two‐dimensional transition‐metal dichalcogenides (TMDCs), organic semiconductors, and perovskite thin sheets (TSs) have contributed to the development of nanoscale photonics, optoelectronics, and valleytronics.^[^ ^1^, ^2^, ^3^, ^4^ ^]^ Various types of exciton species formed in HSs have been intensively studied owing to their distinctive light emission and spin‐dependent characteristics and different binding energies.^[^ ^5^, ^6^, ^7^ ^]^ Recently, interlayer excitons (IXs) with aligned dipoles, that is, bound electron and hole pairs formed at the interface of HSs, have exhibited distinctive light emission characteristics, including long lifetime,^[^ ^8^, ^9^ ^]^ spin and valley dependency,^[^ ^10^, ^11^ ^]^ and gate‐field modulation.^[^ ^12^, ^13^ ^]^ These provide a promising platform for advanced excitonic devices in solar cells, light‐emitting diodes (LEDs), lasers, phototransistors, photodetectors, and quantum information processing.^[^ ^14^, ^15^, ^16^, ^17^ ^]^

IXs were observed in TMDCs‐based HSs, such as MoSe_2_/WSe_2_,^[^ ^5^, ^18^ ^]^ MoS_2_/WSe_2_,^[^ ^12^, ^13^ ^]^ WS_2_/WSe_2_,^[^ ^11^, ^19^ ^]^ and WS_2_/HfS_2_,^[^ ^20^ ^]^ in which the emission energy and diffusion length were controlled by external electric fields through the directional alignment of out‐of‐plane dipole moments.^[^ ^9^, ^12^ ^]^ Recently, Kim et al. reported the photoluminescence (PL) emission of IXs observed in the visible light region (*λ*_em_ = 675–700 nm) using the WS_2_/PbI_2_ HS.^[^ ^21^ ^]^ The IX coupling from the interface of WS_2_/tetracene HSs was studied in terms of the characteristics of charge transfer (CT) excitons from delocalized to localized states (trapping) and vice versa.^[^ ^22^ ^]^ Chen et al. investigated the dynamics of IXs in various perovskite HSs with WSe_2_ using power‐ and temperature‐dependent PL spectra.^[^ ^23^ ^]^

Organic‐inorganic halide perovskites with the structure ABX_3_ (A: organic cations; B: metal cations; X: halide anions) are interesting active materials for optoelectronic devices^[^ ^24^, ^25^, ^26^ ^]^ because of their high PL quantum yield^[^ ^27^, ^28^ ^]^ and long diffusion lengths with long lifetimes.^[^ ^29^, ^30^ ^]^ However, surface defects and degradation of perovskites in the environment cause performance deterioration. Recently, the characteristics of perovskites have been enhanced through hybridization with functionalized quantum dots (QDs) owing to energy transfer (ET) and surface passivation effects.^[^ ^24^, ^31^, ^32^ ^]^ Sanchez et al. reported near‐infrared (NIR) emission at 1240–1400 nm in HSs with MAPbI_3−x_Cl_x_ perovskites and PbS‐CdS‐QDs by adjusting the energy band structure.^[^ ^33^ ^]^ The light emission and absorption characteristics in the far‐red optical range (800–900 nm) for the perovskite‐ and QD‐based HSs can provide an efficient collection of solar energy and can be applied to broad‐range and sensitive photodetectors,^[^ ^32^ ^]^ solar cells,^[^ ^34^ ^]^ and NIR LED for biomedical imaging and optical communication.^[^ ^35^ ^]^

Methylammonium lead iodide (MAPbI_3_) perovskite exhibits the unique property of temperature‐dependent structural phase transitions.^[^ ^36^, ^37^, ^38^, ^39^ ^]^ MAPbI_3_ perovskite shows two PL peaks related to the orthorhombic (O) and tetragonal (T) phases at a phase transition temperature (*T*_c_) of 140 K.^[^ ^36^, ^37^, ^38^, ^39^, ^40^ ^]^ Khatun et al. investigated an unusually large shift in the conduction band minimum (CBM) and valence band maximum (VBM) during the phase transition using scanning tunneling spectroscopy.^[^ ^39^ ^]^ Therefore, the investigation of far‐red IXs characteristics of MAPbI_3_/QDs HS at low temperatures across *T*_c_ is an interesting subject with a correlation of phase transition. In addition, the modulation of energy band alignments (EBAs) of MAPbI_3_‐based HSs from type‐II to type‐I or vice versa depending on the diameter of the QDs is challenging research to study photoinduced CT and ET effects and to apply IXs for excitonic devices. Exploration of the hybrid effects of MAPbI_3_ perovskite with QDs can be a platform for biomedical imaging and future photonics, optoelectronics, and valleytronics.

In this study, HSs of MAPbI_3_ TS and CdSe‐ZnS‐QDs with different diameters were prepared to manipulate EBA. The far‐red IX emission for the MAPbI_3_/CdSe‐ZnS‐QD (645) HS (type‐II EBA) was observed at 1.42 eV (= 873 nm). The lifetime of the far‐red IXs of the HS was estimated to be 5.68 µs at 3 K, which is considerably longer than that of intralayer excitons. The peaks of the far‐red IXs were blue‐shifted with increasing excitation power owing to the screening effect and repulsive interaction of aligned dipoles, whereas those of intralayer excitons showed negligible shifts. The temperature dependence of the PL peaks of the IXs shows abnormal behavior from 60 to 90 K owing to the phase transition of MAPbI_3_. The dipole direction of the far‐red IXs investigated by back focal plane (BFP) mapping was distinctively out‐of‐plane than that of the intralayer excitons. An enhanced photocurrent and detectivity of the MAPbI_3_/CdSe‐ZnS‐QD (645) HS device were observed with excitation at *λ*_ex_ = 810 nm. MAPbI_3_/CdSe‐ZnS‐QD is a promising HS for advanced excitonic devices based on long‐lived IXs in the far‐red range and controllable energy band structure.

## 2 Results and Discussion

### 2.1 Structural and Optical Characteristics

**Figure 1:**
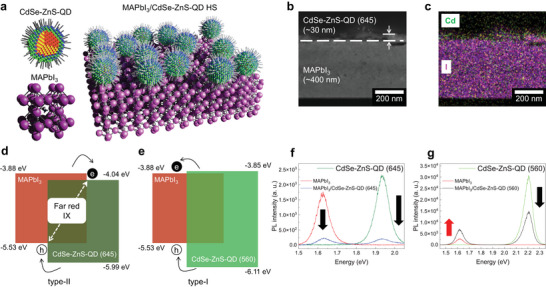
a) Schematic illustration of the MAPbI_3_/CdSe‐ZnS‐QD heterostructure (HS). b) TEM image and c) EDS mapping image of the cross‐sectional view of MAPbI_3_/CdSe‐ZnS‐QD (645) HS. Energy band structures of d) MAPbI_3_/CdSe‐ZnS‐QD (645 nm) HS (type‐II EBA) and e) MAPbI_3_/CdSe‐ZnS‐QD (560 nm) HS (type‐I EBA). f) PL spectra for the CdSe‐ZnS‐QD (645) (green curve), MAPbI_3_ (red curve), and MAPbI_3_/CdSe‐ZnS‐QD (645) HS (blue curve) at 297 K. g) PL spectra for the CdSe‐ZnS‐QD (560) (green curve), MAPbI_3_ (red curve), and MAPbI_3_/CdSe‐ZnS‐QD (560) HS (black curve) at 297 K.

**Figure** **1**a shows a schematic of the MAPbI_3_/CdSe‐ZnS‐QD HS. Figure 1b,c shows the transmission electron microscopy (TEM) image and corresponding energy dispersive X‐ray spectroscopy (EDS) mapping image, respectively, of the cross sections of MAPbI_3_/CdSe‐ZnS‐QD (645) HS. From the TEM image, the thicknesses of the CdSe‐ZnS‐QD (645) and MAPbI_3_ layers were estimated to be ≈30 and 400 nm, respectively. The Cd element (green) from the top layer and the I element (magenta) from the bottom layer confirmed the bilayer formation of HS. The TEM images obtained from the different batches of QDs (645) were measured to confirm the bilayer HS, in which the thickness of the CdSe‐ZnS‐QD (645) layer was ≈40 nm (Figure S1, Supporting Information). The diameters of QDs used in this study were directly measured by TEM, which showed the high uniformity (Figure S2, Supporting Information).

Figure 1d shows the type‐II EBA by MAPbI_3_/CdSe‐ZnS‐QD (645) HS with the formation of IXs through photoinduced CT, and Figure 1e shows the type‐I EBA by MAPbI_3_/CdSe‐ZnS‐QD (560) HS. The meaning of 645 in the parentheses of MAPbI_3_/CdSe‐ZnS‐QD (645) is the wavelength of the emission peak of the CdSe‐ZnS‐QD. The 560 in the parentheses of MAPbI_3_/CdSe‐ZnS‐QD (560) is the wavelength of the emission peak of the corresponding QD. The VBM of MAPbI_3_, CdSe‐ZnS‐QD (645), and CdSe‐ZnS‐QD (560) were estimated to be ≈−5.53, −5.99, and −6.11 eV, respectively, as obtained from the UPS spectra (Figure S3, Supporting Information). The CBM of the MAPbI_3_, CdSe‐ZnS‐QD (645), and CdSe‐ZnS‐QD (560) were evaluated to be ≈−3.88, −4.04, and −3.85 eV, respectively, using optical absorbance and PL spectra of the corresponding samples (Figure S4, Supporting Information). It is noted that the energy band gap (*E*_g_) of the QDs is determined by the diameter of QDs based on quantum confinement effect. With decreasing the diameter of QDs, the *E*_g_ increases and the emission is blue‐shifted. Therefore, the diameters of QDs affect the type‐II and ‐I EBAs for the HS with MAPbI_3_ as shown in Figure 1d,e. The VBM and CBM of the QDs and the EBAs of the HSs with QDs can be controlled by the diameter of the QDs.

Figure 1f shows the PL spectra of CdSe‐ZnS‐QD (645) (green curve), MAPbI_3_ (red curve), and MAPbI_3_/CdSe‐ZnS‐QD (645) (blue curve) at room temperature (RT = 297 K). It should be noted that the excitation power (10 µW) of the incident laser for MAPbI_3_/CdSe‐ZnS‐QD (645) HS was 100 times greater than that (100 nW) of pristine CdSe‐ZnS‐QD (645) and MAPbI_3_. The characteristic PL peaks of the CdSe‐ZnS‐QD (645) and the MAPbI_3_ were observed at 1.93 and 1.63 eV, respectively. Notably, the PL intensities of both CdSe‐ZnS‐QD (645) and MAPbI_3_ drastically decreased (i.e., quenching) after the formation of HS owing to the photoinduced CT effect across the heterointerface. Figure 1g shows the PL spectra of the CdSe‐ZnS‐QD (560) (green curve), MAPbI_3_ (red curve), and MAPbI_3_/CdSe‐ZnS‐QD (560) (black curve) excited at the power of 100 nW. The characteristic PL peaks of the CdSe‐ZnS‐QD (560) and the MAPbI_3_ were observed at 2.21 and 1.63 eV, respectively. The enhancement of the PL intensity of MAPbI_3_ by 2.5 times was observed after hybridization with CdSe‐ZnS‐QD (560) due to the ET effect in type‐I EBA.

### 2.2 Photoluminescence and Exciton Dynamics at Low Temperatures

**Figure 2:**
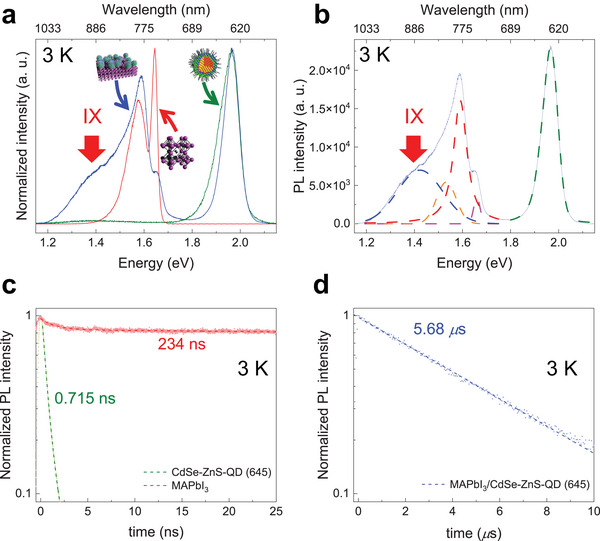
a) Normalized PL spectra of the CdSe‐ZnS‐QD (645) (green curve), MAPbI_3_ (red curve) and their HS (blue curve) at 3 K. b) PL spectra of the MAPbI_3_/CdSe‐ZnS‐QD (645) HS at 3 K with five deconvoluted curves: CdSe‐ZnS‐QD (645) (green dashed curve; 1.97 eV = 629 nm), MAPbI_3_‐O (magenta dashed curve; 1.66 eV = 747 nm), MAPbI_3_‐T (red dashed curve; 1.59 eV = 780 nm), MAPbI_3_‐D (orange dashed curve; 1.53 eV = 810 nm), and IXs (blue dashed curve; 1.42 eV = 873 nm). Time‐resolved PL (tr‐PL) spectra c) for the CdSe‐ZnS‐QD (645) (green curve) and MAPbI_3_ (red curve) and that d) for the IXs (blue curve) at 3 K.

**Figure** **2**a shows the normalized PL spectra of CdSe‐ZnS‐QD (645) (green curve), MAPbI_3_ (red curve), and MAPbI_3_/CdSe‐ZnS‐QD (645) (blue curve) HS at 3 K. The PL characteristic peaks of the pristine QD and MAPbI_3_ in the MAPbI_3_/CdSe‐ZnS‐QD (645) HS were observed at 1.93 and 1.63 eV, respectively (Figure 2a). Interestingly, a new broad PL shoulder signal for HS (blue curve) was observed at 1.42 eV (Figure 2a). The PL spectrum of the MAPbI_3_ only was deconvoluted into three peaks corresponding to the orthorhombic (MAPbI_3_‐O), tetragonal (MAPbI_3_‐T), and defect (MAPbI_3_‐D) phases (Figure S5, Supporting Information) as previously reported.^[^ ^38^, ^41^, ^42^ ^]^ To determine the characteristics of the PL emission, the PL spectrum from the HS was deconvoluted into five characteristic curves, as shown in Figure 2b. The PL peak centered at 1.97 eV (green curve) originates from the CdSe‐ZnS‐QD (645), and other three PL peaks at 1.66, 1.59, and 1.53 eV are due to the orthorhombic (magenta curve), tetragonal (red curve), and defect (orange curve) phases of MAPbI_3_, respectively. Notably, the PL peak centered at 1.42 eV originates from the far‐red IXs through the photoinduced CT effect between the MAPbI_3_ and CdSe‐ZnS‐QD (645) layers (type‐II EBA). The far‐red IXs for HS were somewhat weaker but still distinctively observed at 50 K, as shown in Figure S6 (Supporting Information).

To investigate the exciton dynamics, time‐resolved PL (tr‐PL) spectra were measured for CdSe‐ZnS‐QD (645), MAPbI_3_, and MAPbI_3_/CdSe‐ZnS‐QD (645) HS at 3 K. As shown in Figure 2c (and Figure S7, Supporting Information), the average lifetimes of excitons (*τ*_avg_) of the CdSe‐ZnS‐QD (645) and MAPbI_3_ were estimated to be ≈0.715 and 234 ns, respectively, obtained from the bi‐exponential decay model (Table S1, Supporting Information). Notably, *τ*_avg_ of the far‐red IXs of the HS was estimated to be ≈5.68 µs (Figure 2d), which is considerably longer (24.3 times) than that (234 ns) of the intralayer excitons of MAPbI_3_ and ultra‐longer (≈7950 times) than that (0.715 ns) of CdSe‐ZnS‐QD (645). The ultra‐long lifetime is a typical characteristic of IXs, which results from the small wave function overlap between the spatially separated electrons and holes in opposite heterolayers and the weak oscillator strength of IXs.^[^ ^8^, ^43^, ^44^ ^]^ The observed ultralong‐lived far‐red IXs play an important role in increasing the efficiency of photodetectors, solar cells, and NIR LEDs.

In CdSe‐ZnS‐QD, the ZnS shell acts as the charge transfer barrier between CdSe core and MAPbI_3_ therefore the thickness of ZnS shell has a crucial effect to the efficiency of CT and IX formation in MAPbI_3_/CdSe‐ZnS‐QD (645) HS. From the TEM image of CdSe‐ZnS‐QDs (645), the ZnS shell thickness was estimated to be 0.1–0.5 nm (Section S2, Supporting Information), which was thin enough to ensure the efficient CT.^[^ ^45^, ^46^ ^]^ The average lifetimes (*τ*_avg_) of pristine CdSe‐ZnS‐QDs (645) and HS systems were also obtained from tr‐PL spectra at 3 K (*P*_in_ = 1 µW), which were estimated to be *τ*_QDpristine_ = 1.96 ns and *τ*_QDHS_ = 0.707 ns, respectively (Figure S8a, Supporting Information). Using the equation for CT efficiency, *E*_HT_ = 1−*τ*_QDHS_/*τ*_QDpristine_,^[^ ^45^, ^46^ ^]^ the CT efficiency (*E*_HT_) was estimated to be ≈64.0%, confirming the efficient CT between CdSe‐ZnS‐QDs (645) and MAPbI_3_ required for IX formation.

**Figure 3:**
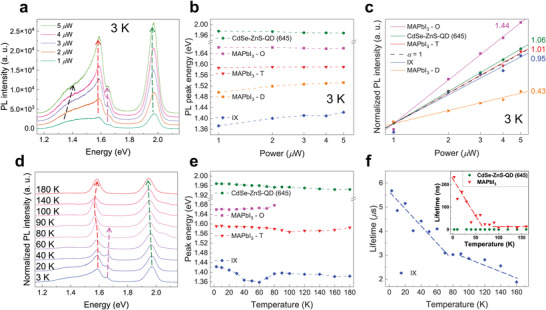
a) PL spectra of MAPbI_3_/CdSe‐ZnS‐QD (645) HS with various excitation powers from 1 to 5 µW. The arrows are eye guidance for the PL peak shift of CdSe‐ZnS‐QD (645) (green), MAPbI_3_‐O (magenta), MAPbI_3_‐T (red), and IXs (black), respectively. Incident excitation power‐dependent b) PL peak position and c) normalized PL intensity for the CdSe‐ZnS‐QD (645) (green), MAPbI_3_‐O (magenta), MAPbI_3_‐T (red), MAPbI_3_‐D (orange), and the HS for IXs (blue) at 3 K. Black dashed curve in c) is *α* = 1 for comparison. d) Normalized PL spectra of MAPbI_3_/CdSe‐ZnS‐QD (645) HS at various temperatures from 3 to 180 K. The arrows are eye guidance for peak shift of CdSe‐ZnS‐QD (645) (green), MAPbI_3_‐O (magenta), and MAPbI_3_‐T (red). e) PL peak energy as a function of temperature for CdSe‐ZnS‐QD (645) (green), MAPbI_3_‐O (magenta), MAPbI_3_‐T (red), and IXs (blue) obtained from the HS. f) Average lifetimes (*τ*_avg_) of the IXs (blue) of the HS as a function of temperature in linear scale. Inset: *τ*_avg_ of excitons of MAPbI_3_ (red) and CdSe‐ZnS‐QD (645) (green) as a function of temperature in linear scale.

The excitation power dependence of the PL spectra of MAPbI_3_/CdSe‐ZnS‐QD (645) HS was measured at 3 K, as shown in **Figure** **3**a. The excitation laser power varied from 1 to 5 µW. The intensities of all PL characteristic peaks for MAPbI_3_/CdSe‐ZnS‐QD (645) HS monotonically increased with increasing excitation power at 3 K (Figure 3a). Figure S9 (Supporting Information) shows the magnification of the power‐dependent PL spectra in the energy range of 1.15–1.5 eV at 3 K, focusing on the far‐red IXs characteristics, and the deconvoluted curves at each excitation power in the full range. With increasing excitation power, the PL peaks of the CdSe‐ZnS‐QD (645), MAPbI_3_‐O, and MAPbI_3_‐T were almost constant (Figure 3b). Notably, with increasing excitation power, the PL peak of the far‐red IXs centered at 1.37 eV (for 1 µW) was blue‐shifted by up to 50 meV (for 5 µW). Because the electrons and holes of IXs are separately located across the HS interface, all IXs naturally possess a permanent dipole direction perpendicular to the HS interface, resulting in dipole–dipole repulsive interactions among IXs,^[^ ^5^, ^9^ ^]^ causing the PL peak of IXs to shift to higher energy with increasing density of IXs. Therefore, the blue shift of the PL peak corresponding to the far‐red IXs with increasing excitation power is consistent with the repulsive dipole–dipole interaction of IXs formed at the heterointerface between MAPbI_3_ and CdSe‐ZnS‐QD (645). In addition, the PL intensity as a function of excitation power can be fitted with the power law *I*_PL_ ∝ (*P*_in_)^*α*^, where *I*_PL_, *P*_in_, and *α* are the PL intensity, excitation power, and exponential factor, respectively. *α* is related to the recombination process of the PL emission, which has values between 1 and 2 corresponding to band‐to‐band transitions and below 1 corresponding to donor‐acceptor and free‐to‐bound transitions.^[^ ^47^ ^]^ The values of *α* are estimated to be 1.06, 1.44, and 1.01 for the intralayer excitons of CdSe‐ZnS‐QD (645), MAPbI_3_‐O, and MAPbI_3_‐T, respectively, as shown in Figure 3c. Notably, the value of *α* for the far‐red IXs is estimated to be 0.95, which lies somewhat high within the reported range of *α* for other IXs (0.6 to 0.9),^[^ ^20^, ^23^ ^]^ implying the high accumulation of charges at the interface of our HS through the effective CT. The high *α* is consistent with the relatively strong blue shift of the PL peak of the far‐red IXs (Figure 3b) and the PL quenching of MAPbI_3_ and CdSe‐ZnS‐QD (645) after the formation of HSs (Figure 1f). Therefore, the excitation power dependence of the far‐red IXs formed in MAPbI_3_/CdSe‐ZnS‐QD (645) HS is clearly distinct from that of intralayer excitons, mainly due to the relatively weak binding energy (15.3 meV) of the IXs (Figure S10, Supporting Information). In addition, as shown in Figure 3b, PL peak shift of MAPbI_3_‐D was ≈30 meV (from 1.50 to 1.53 eV) as the input power increased from 1 to 5 µW, notably smaller than that of the IX one (50 meV). In Figure 3c, the *α* of the MAPbI_3_‐D was estimated to be 0.43, and such low value is typical characteristics of defect states. The IXs display very different spectral characteristics from those of defect emission, such as strong blue‐shift and near‐linear power slope with increasing excitation intensity.

The dynamics and interactions of the IXs related to the phase transition of MAPbI_3_/CdSe‐ZnS‐QD (645) HS were investigated via temperature‐dependent PL and tr‐PL spectra obtained in the range of 3–180 K. The normalized PL spectra of MAPbI_3_/CdSe‐ZnS‐QD (645) HS at various low temperatures (3–180 K) are shown in Figure 3d. As seen in the PL spectra of MAPbI_3_/CdSe‐ZnS‐QD (645) HS, the PL peak corresponding to CdSe‐ZnS‐QD (645) at 1.97 eV (3 K) was gradually red‐shifted as the temperature increased (green dashed arrow in Figure 3d) because of the enhanced electron–phonon interaction and lattice expansion.^[^ ^48^, ^49^ ^]^ The temperature‐dependent behaviors of the PL peaks corresponding to the MAPbI_3_‐O and MAPbI_3_‐T phases are similar to those reported previously.^[^ ^36^, ^42^, ^50^, ^51^ ^]^ The PL peak positions (1.59 eV) of MAPbI_3_‐T were almost constant with varying temperature (Figure 3e). Interestingly, for MAPbI_3_/CdSe‐ZnS‐QD (645) HS, the characteristic PL peaks due to the orthorhombic phase of MAPbI_3_ (MAPbI_3_‐O) were observed from the phase transition temperature *T*_c_ = 90 to 3 K (Figure 3e). It should be noted that *T*_c_ = 90 K of HS was lower than that (140 K) of pristine MAPbI_3_ (Figure S11, Supporting Information). This result implies that *T*_c_ from the tetragonal to orthorhombic phase decreased from 140 to 90 K after hybridization with the CdSe‐ZnS‐QD (645). According to a previous report,^[^ ^50^ ^]^ a change in the concentration of defects can induce a variation in *T*_c_ of the MAPbI_3_. X‐ray photoelectron spectroscopy (XPS) was performed to identify the origin of the variation in *T*_c_. The Pb 4f_5/2_ binding energy decreased by ≈100 meV after hybridization with QD (Figure S12, Supporting Information), suggesting that the defect states of MAPbI_3_ were reduced through surface passivation after hybridization with QD (645).^[^ ^24^, ^31^, ^32^ ^]^ Therefore, the reduced *T*_c_ from 140 to 90 K for MAPbI_3_ (Figure S13, Supporting Information) is attributed to the hybridization and passivation effects of the CdSe‐ZnS‐QDs (645). Notably, the fluctuation of the PL peak position, that is, the change in the slope of temperature‐dependent PL peaks for the IXs, was observed near and below *T*_c_ = 90 K of the MAPbI_3_/CdSe‐ZnS‐QD (645) HS, as shown in Figure 3e, which can be explained in terms of the structural phase transition of MAPbI_3_ and the fact that the emission characteristics of the IXs are determined by the energy band structures of the constituent layers of the HS. In other words, the abnormal variation in the PL peak of the far‐red IXs below *T*_c_ (from 90 to 60 K) is related to the rapid change in the CBM and VBM of MAPbI_3_ owing to the structural phase transition. The pristine MAPbI_3_ goes under the phase transition, the band edges of MAPbI_3_ show abrupt energy shifts (≈100 meV for VBM and 150 meV for CBM below 140 K) near the *T*_c_ by the overlap reduction of lead and iodine orbitals.^[^ ^39^ ^]^ Therefore, because of the VBM shift of MAPbI_3_ affected by the phase transition from tetragonal to orthorhombic structure, the IX emission energy was ≈1.4 eV in the low temperature regime, consistent with the observation in Figure 2b. Further details are provided in Figure S14 (Supporting Information).

The PL peak energy of the far‐red IXs increased as the temperature decreased from 60 to 3 K, owing to the temperature‐induced band shift and screening effect of the transferred and accumulated charges at the interface of the HS. The effective CT between MAPbI_3_ and CdSe‐ZnS‐QD (645) became more active at lower temperatures based on type‐II EBA, resulting in an increase in the density of IXs. The results suggest that the far‐red IXs of the MAPbI_3_/CdSe‐ZnS‐QD (645) HS were formed through CT in type‐II EBA and were affected by the phase transition of the MAPbI_3_ layer at specific temperatures (below 90 K). Our results exhibit distinctive power‐ and temperature‐dependent behaviors of the far‐red IXs of MAPbI_3_/CdSe‐ZnS‐QD (645) HS, which are affected by the structural phase transition of perovskite MAPbI_3_, and are clearly distinguished from the intralayer excitons of pristine MAPbI_3_ and CdSe‐ZnS‐QD.

Figure 3f shows the *τ*_avg_ of intralayer excitons and far‐red IXs of MAPbI_3_/CdSe‐ZnS‐QD (645) HS as a function of temperature in linear scale. The *τ*_avg_ of CdSe‐ZnS‐QD (645) (green markers) was almost constant at 0.60 ns in the range of 3–180 K; however, the *τ*_avg_ of MAPbI_3_ decreased from 234 ns (3 K) to 14.6 ns (160 K) (red markers) as shown in the inset of Figure 3f. The slope of temperature dependence of *τ*_avg_ for MAPbI_3_ was changed at 80–90 K (near *T*_c_) as shown in the inset of Figure 3f. Notably, the slope of temperature dependence of *τ*_avg_ of the far‐red IXs was also changed at 80–90 K (Figure 3f). Therefore, the phase transition of MAPbI_3_ affects the temperature dependence of *τ*_avg_ of the far‐red IXs. The *τ*_avg_ of the far‐red IXs decreased with increasing temperature from 5.68 µs at 3 K to 1.73 µs at 180 K. The observed ultra‐long lifetime of IXs is the most important property for the application of excitonic devices.^[^ ^8^, ^33^, ^52^ ^]^

### 2.3 Dipole Characteristics

**Figure 4:**
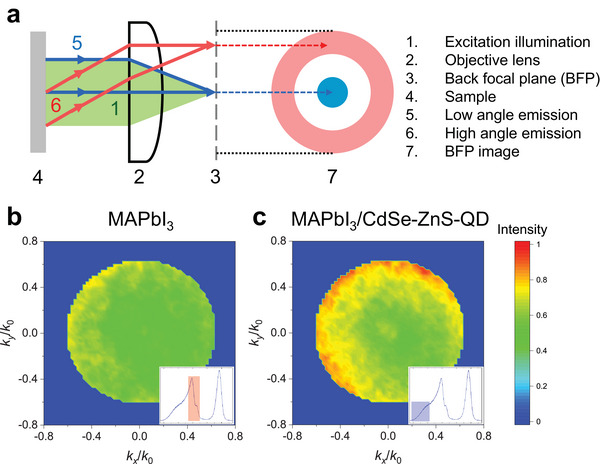
a) Schematic of optical layout of the back focal plane (BFP) imaging. The angle of the emission determines the radial distance from the center on BFP image. BFP mapping images of b) MAPbI_3_ and c) MAPbI_3_/CdSe‐ZnS‐QD (645) HS for IXs normalized using the image of CdSe‐ZnS‐QD as a reference with normalized scale [0,1]. The spectral ranges chosen for MAPbI_3_ (inset of (b)) and IXs of HS (inset of (c)).

To investigate the dipole characteristics of far‐red IXs and intralayer excitons, *k*‐space emission patterns that resolve the PL emission angle were obtained from BFP imaging. **Figure** **4**a shows the optical setup for BFP imaging, where the PL emission angles are mapped to the BFP. Thus, the axes (*k*_x_/*k*_0_ and *k*_y_/*k*_0_) of the BFP images in Figure 4b,c represent the in‐plane components (sin *θ*_em_) of the wavevector of the emission photons with an emission angle *θ*_em_ with respect to the optical axis.^[^ ^18^, ^53^, ^54^ ^]^ A series of mapping images was acquired using a rotating linear polarizer in front of the detector for every 30° from 0° to 330°, and these BFP images were averaged to obtain a high signal‐to‐noise ratio. The BFP mapping images of the MAPbI_3_ emission and the far‐red IX emission of MAPbI_3_/CdSe‐ZnS‐QD (645) are shown in Figure 4b,c, respectively. The insets indicate the spectral ranges chosen for MAPbI_3_ and IXs of HS. Both BFP mapping images were normalized using the BFP image of CdSe‐ZnS‐QD as the reference signal to compensate for the errors that can be caused by drift and fluctuation of optical alignments, considering that the QDs should have an isotropic emission pattern owing to the random directions of the dipole moments of the excitons. The BFP image of MAPbI_3_ shows quite uniform intensities through the BFP plane (Figure 4b), whereas that of the IXs shows a relatively stronger emission at the edge than at the center (Figure 4c), indicating that the out‐of‐plane components of the exciton dipole moment are considerably larger for IXs than for the intralayer excitons of CdSe‐ZnS‐QD (645) or MAPbI_3_. The slight gradient along the diagonal of the BFP image is believed to originate from the imperfect alignment of the scanning plane during BFP mapping. The observed intense edge pattern of the BFP image of the far‐red IXs is consistent with previous reports on other IXs.^[^ ^18^ ^]^ Combined with the power‐dependent characteristics of IXs, the results indicate that the dipoles of the far‐red IXs are aligned and directed toward the out‐of‐plane direction, which strongly confirms the presence of IXs at the heterointerface of MAPbI_3_ and CdSe‐ZnS‐QD HS.

### 2.4 Photodetectors

**Figure 5:**
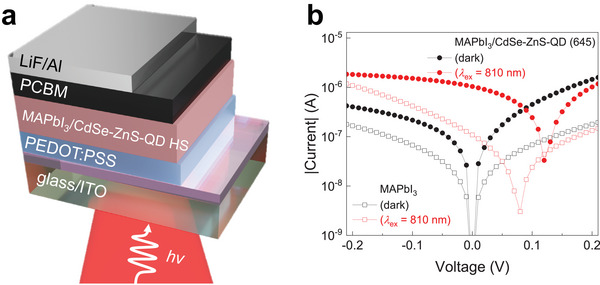
a) Schematic of the photodetector with vertical structure. b) Current–voltage characteristic curves (*I* vs *V*) of pristine MAPbI_3_ (open markers) and MAPbI_3_/CdSe‐ZnS‐QD (645) HS (solid markers) photodetectors in the dark (black) and light‐irradiation condition with *λ*_ex_ = 810 nm (red) (power = 0.1 mW).

The type‐II EBA to form IXs in MAPbI_3_/CdSe‐ZnS‐QD (645) HS provides a promising pathway for optoelectronics and photonics operating in the NIR range (*λ* ≥ 700 nm). Photodetectors using pristine MAPbI_3_ and MAPbI_3_/CdSe‐ZnS‐QD (645) HS were fabricated with a transparent ITO electrode, [6,6]‐Phenyl‐C61‐butyric acid methyl ester (PCBM) electron transport layer, and PEDOT:PSS hole transport layer, as shown in **Figure** **5**a. The current *I* of the pristine MAPbI_3_ (open markers) photodetectors increased from 3.81 × 10^−8^ A (in the dark) to 2.61 × 10^−7^ A (with *λ*_ex_ = 810 nm; open red markers) at a bias (*V*_d_) of −0.06 V. The *I* of the MAPbI_3_/CdSe‐ZnS‐QD (645) HS photodetectors (solid markers) increased from 1.39 × 10^−7^ A (in the dark condition) to 1.36 × 10^−6^ A (with *λ*_ex_ = 810 nm; solid red markers) at *V*_d_ = −0.06 V, as shown in Figure 5b. It should be noted that the incident LED with *λ*_ex_ = 810 nm can directly excite the far‐red IXs in the MAPbI_3_/CdSe‐ZnS‐QD (645) HS. The photocurrent of MAPbI_3_ was drastically enhanced, and the open‐circuit–voltage was positively shifted, implying an enhanced photovoltaic effect in the far‐red region through hybridization with CdSe‐ZnS‐QD. *I*–*V* characteristic curves at different wavelengths of the incident light irradiation are shown in Figure S15 (Supporting Information). The detectivity (*D* ^*^) for the device performance can be expressed as follows:^[^ ^55^, ^56^ ^]^

(1) $$D^{*}=\frac{\left(I_{\mathrm{light}}-I_{\mathrm{dark}}\right)}{P\cdot A\cdot \sqrt{\frac{4k_{B}T}{R_{0}A}\;+\frac{2eI_{\mathrm{dark}}}{A}}}\;$$

Here, *I*_light_ and *I*_dark_ are the currents with and without light irradiation, respectively; *P* and *A* are the power density and effective area, respectively; and *k*_B_, *T*, *R*_0_, and *e* are the Boltzmann constant, temperature, resistance, and elementary charge, respectively. Notably, with *λ*_ex_ = 810 nm (= 1.53 eV) close to the far‐red IX energy gap (1.49 eV) of the HSs, the values of *D* ^*^ for the MAPbI_3_ photodetectors increased from 8.8 × 10^9^ to 7.37 × 10^10^ Jones (8.4 times) after the hybridization with CdSe‐ZnS‐QD (645). Interestingly, the enhancement of *D* ^*^ using MAPbI_3_/CdSe‐ZnS‐QD (645) HS photodetectors was measured at an extremely low bias of −0.06 V. The low‐voltage performance can lead to highly sensitive photodetectors owing to the low‐noise dark current. Therefore, the results suggest that the far‐red IXs and their dissociation in MAPbI_3_/CdSe‐ZnS‐QD (645) HS contributed to the enhancement of *D* ^*^ with low‐voltage operation.

## 3 Conclusion

The HSs of MAPbI_3_/CdSe‐ZnS‐QD (645) and MAPbI_3_/CdSe‐ZnS‐QD (560) with type‐II and type‐I EBAs, respectively, were fabricated to study IXs. Far‐red PL emission for MAPbI_3_/CdSe‐ZnS‐QD (645) HS was observed at *λ*_em_ = 873 nm (1.42 eV) at 3 K. The average lifetime of PL emission of the far‐red IXs from the MAPbI_3_/CdSe‐ZnS‐QD (645) HS was estimated to be 5.68 µs, which was ultra‐longer than the 0.715 ns of the CdSe‐ZnS‐QD (645). The PL peak energy for the IXs was blue‐shifted with increasing excitation power owing to the screening effect of the transferred and accumulated charges at the HS interface. Abnormal behavior of the temperature‐dependent PL characteristics of the IXs was observed below 90 K owing to the structural phase transition of MAPbI_3_. The dipole direction of the far‐red IXs of MAPbI_3_/CdSe‐ZnS‐QD (645) HS obtained from the BFP mapping images was strongly out‐of‐plane and clearly distinguishable from those of the intralayer excitons (MAPbI_3_ and CdSe‐ZnS‐QD (645)). In type‐II EBA, the far‐red IXs and their dissociation contributed to the increase in photocurrent and detectivity, which were applied to broad‐band excitonic devices. Our results and analysis can be used as a platform for understanding the characteristics of IXs in perovskite/QD HSs, leading to new developments in IX‐based optoelectronics, photonics, and quantum information processors.

## 4 Experimental Section

### Fabrication of MAPbI_3_/CdSe‐ZnS‐QD Heterostructures

Methylammonium lead iodide (MAPbI_3_) perovskite precursor solution was prepared by dissolving methylammonium iodide (CH_3_NH_3_I; MAI, 3.18 g) and lead iodide (PbI_2_, 9.22 g) powder with the same molar ratio in *N*,*N*‐dimethylformamide (DMF, 20 mL). The solution was stirred for 24 h after sonication for 1 h and filtered (poly(1,1,2,2‐tetrafluoroethylene); PTFE, 0.25 µm). The octadecylamine functionalized CdSe‐ZnS core‐shell QDs (*λ*_em_ = 645 nm) (denoted as CdSe‐ZnS‐QD (645)) were dispersed in toluene at a density of 0.1 mg mL^−1^. The solution was stirred for 24 h and then sonicated for 1 h. An oleic acid functionalized CdSe‐ZnS QDs (*λ*_em_ = 560 nm) (denoted as CdSe‐ZnS‐QD (560)) solution was also prepared in toluene. All reagents were purchased from Sigma‐Aldrich. The left image in Figure 1a shows the schematic of the chemical structure of the CdSe‐ZnS core‐shell QD and MAPbI_3_.

To fabricate MAPbI_3_ TS, anti‐solvent method was used.^[^ ^57^ ^]^ The MAPbI_3_ solution was spin‐coated onto the Si/SiO_2_ substrate at 4000 rpm for 25 s and treated with anisole as an anti‐solvent for 10 s. The MAPbI_3_ TS was annealed at 100 °C for 5 min before further processing. The CdSe‐ZnS‐QD solution was drop‐cast onto the surface of MAPbI_3_ and annealed at 100 °C for 30 min. The thicknesses of the MAPbI_3_ and CdSe‐ZnS‐QD (645) layers for optical characteristics were measured to be ≈400 and 30 nm, respectively, which was confirmed by cross‐sectional TEM analysis.

For fabrication of MAPbI_3_‐based photodetectors, patterned ITO glass was used as the electrode. For the hole transport layer, the PEDOT:PSS solution (CH 8000; Clevios) was dually spin‐coated onto ITO at 3000 rpm for 5 s after 600 rpm for 5 s and dried at 155 °C for 15 min. MAPbI_3_/CdSe‐ZnS‐QD (645) HS was fabricated on ITO/PEDOT:PSS under the same conditions as the optical device. Subsequently, for the electron transport layer, the PCBM solution was spin coated at 1800 rpm for 30 s and dried at 100 °C for 10 min. A LiF/Al top electrode was deposited via thermal evaporation. A schematic of the device is shown in Figure 5a.

### Measurements

The TEM images and corresponding EDS mapping images were obtained using a Tecnai (FEI company). The PL spectra were measured using a homemade high‐resolution laser confocal microscope (LCM) coupled with a spectrometer (Acton SpectraPro 300i; Princeton Instruments) and a charge‐coupled device (PIXIS 100; Princeton Instruments) at low temperatures (3–297 K) using a closed‐loop cryostat (Cryostation; Montana Instruments). To measure tr‐PL spectra, time‐correlated single photon counting (TCSPC) system (Simple‐Tau; Becker & Hickl GmbH) and a silicon avalanche photodiode (SPCM‐AQRH‐14‐FC; Excelitas Technologies) were used. The same optical system, including a sync signal generator module (LSG‐02; Becker & Hickl GmbH) and a pulse generator card (DDG‐210; Becker & Hickl GmbH), was used to measure µs‐scale tr‐PL spectra. Short‐pass (SP) or long‐pass (LP) filters for the tr‐PL spectra were used to collect only signals originating from the CdSe‐ZnS‐QD (645) (SP 650 nm; over 1.91 eV), MAPbI_3_ (LP 750 and SP 800 nm; between 1.55 and 1.65 eV), or IXs (LP 850 nm; under 1.46 eV). The excitation source for the PL and tr‐PL spectra was a ps‐diode laser (BDL‐488‐SMN; Becker & Hickl GmbH) with a continuous wave and 20‐MHz‐repetition mode, respectively. BFP mapping patterns were measured using the same LCM system with a custom‐made scanning BFP setup.^[^ ^54^ ^]^ Ultraviolet photoelectron spectroscopy (UPS) and XPS were performed using a Nexsa system (Thermo Scientific). The absorption spectra were measured using a UV/vis spectrometer (Agilent 8453). Current–voltage (*I*–*V*) characteristic curves were measured using a source measurement unit (2634 B system Sourcemeter SMU; Keithley) with and without the use of an 810 nm collimated LED (M810L3‐C4; Thorlabs).

## Conflict of Interest

The authors declare no conflict of interest.

## Supporting information

Supporting Information

## Data Availability

The data that support the findings of this study are available from the corresponding author upon reasonable request.
