# Supplementary material for: Far‐Red Interlayer Excitons of Perovskite/Quantum‐Dot Heterostructures
Source: Adv Sci (Weinh). 2023 Mar 20;10(14):2207653. doi: 10.1002/advs.202207653 (PMC10190583; doi:10.1002/advs.202207653)
Supplement: Supplementary file 1 — Supporting Information [file ADVS-10-2207653-s001.pdf]

ADVANCED SCIENCE
Open Access

Supporting Information

for *Adv. Sci.*, DOI 10.1002/advs.202207653

Far-Red Interlayer Excitons of Perovskite/Quantum-Dot Heterostructures

Taek Joon Kim, Sang-hun Lee, Eunji Lee, Changwon Seo, Jeongyong Kim and Jinsoo Joo**

Supporting Information

Far-Red Interlayer Excitons of Perovskite/Quantum-Dot Heterostructures

Taek Joon Kim, Sang-hun Lee, Eunji Lee, Changwon Seo, Jeongyong Kim, and Jinsoo Joo**

Section S1. TEM image and EDS mapping image of different batch of $MAPbI_3$/CdSe-ZnS-QD (645) heterostructure

Figure S1a and b show TEM image and EDS mapping image, respectively, of cross-sectional view of different batch of $MAPbI_3$/CdSe-ZnS-QD (645) HS. The thickness of CdSe-ZnS-QD (645) layer was evaluated to be approximately 40 nm.

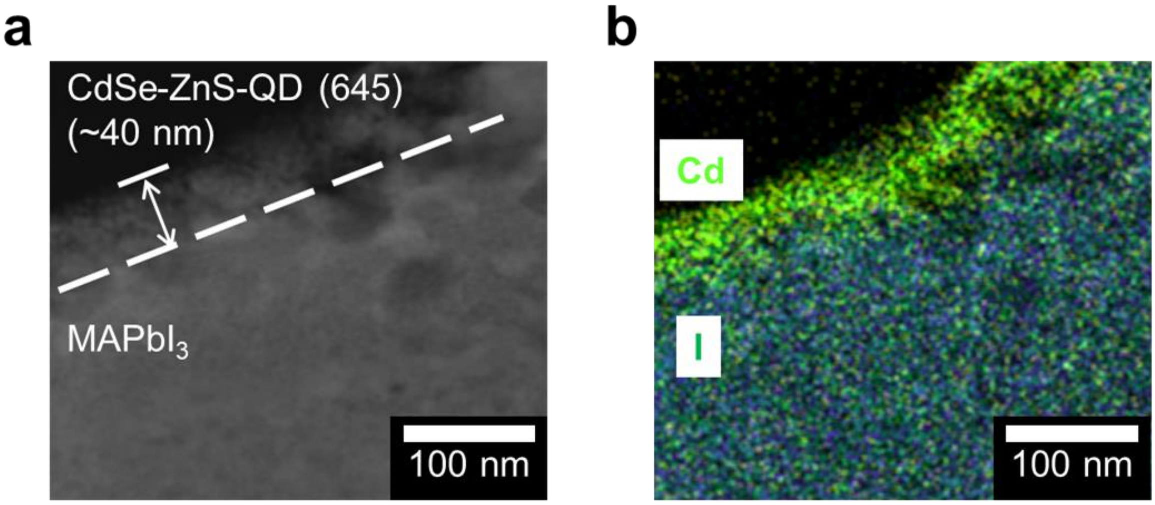

Figure S1. a) TEM image and b) EDS mapping image of the cross-section of different batch of $MAPbI_3$/CdSe-ZnS-QD (645) HS. Cd (light green) and I (dark green) elements indicate the existence of the CdSe-ZnS-QD (645) and $MAPbI_3$, respectively.

Section S2. TEM image and size distribution of CdSe-ZnS-QD (645) and CdSe-ZnS-QD (560)

Figure S2a and b show TEM image and histogram for the diameter of CdSe-ZnS-QD (645), respectively. Figure S2c and d show TEM image and histogram for the diameter of CdSe-ZnS-QD (560), respectively. Average diameters (d_{mean}) of QDs were estimated to be 7.6 ± 1.5 nm for CdSe-ZnS-QD (645) and to be 9.5 ± 0.66 nm for CdSe-ZnS-QD (560), as shown in Figure S2b and d. The diameters of core of CdSe-ZnS-QDs (645) and that of CdSe-ZnS-QD (560) were estimated to be 6.7 – 7.5 nm and 4.6 – 5.0 nm, respectively, using Brus equation.[1]

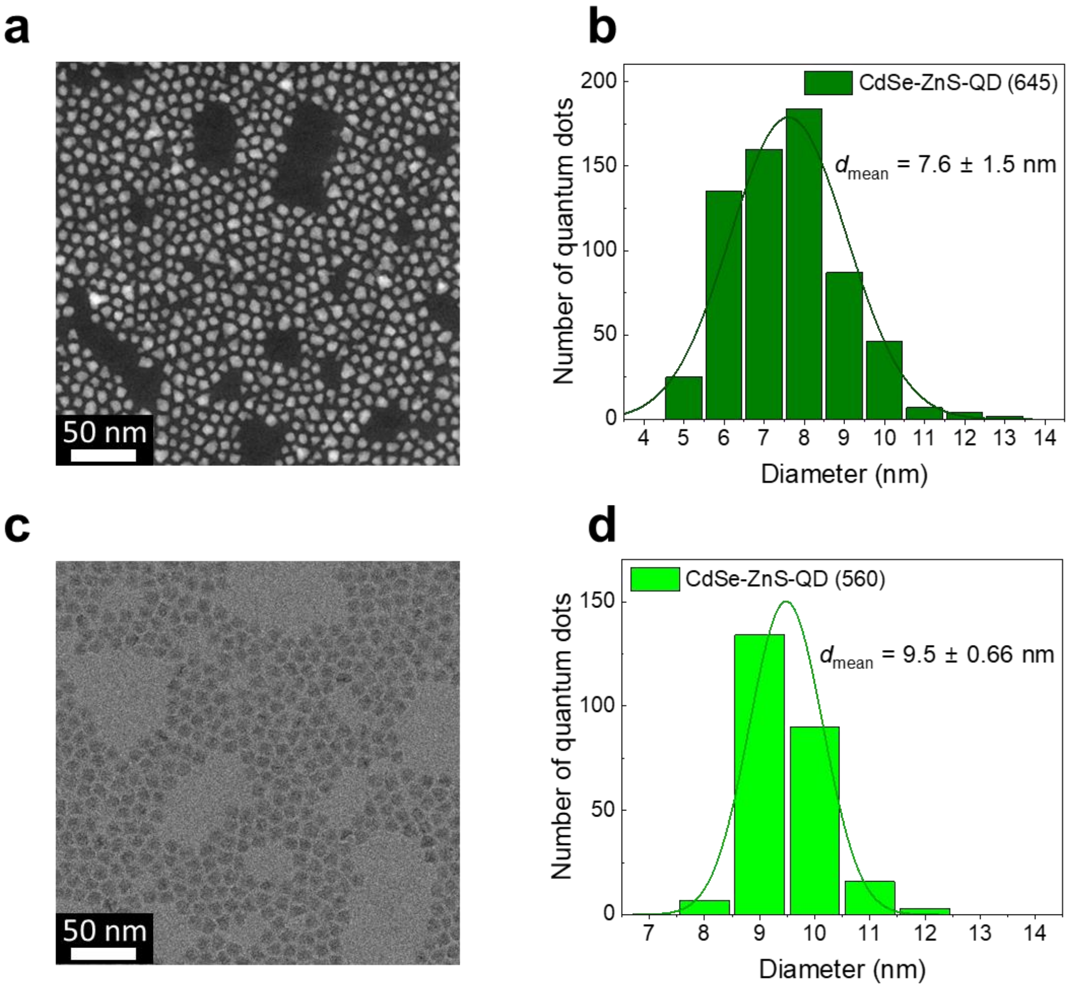

Figure S2. a) TEM image and b) histogram for size distribution of CdSe-ZnS-QDs (645). c) TEM image and d) histogram for size distribution of CdSe-ZnS-QDs (560).

Section S3. UPS spectra for the $MAPbI_3$, CdSe-ZnS-QD (645), and CdSe-ZnS-QD (560)

From the ultraviolet photoelectron spectroscopy (UPS) spectra, the energy of the valence band maximum (VBM) can be estimated as follows:

$$E_{VBM} = h\nu - E_{cutoff} + E_{rel}, \qquad \text{(S1)}$$

where E_{VBM}, $h\nu$, E_{cutoff}, and E_{rel} are the VBM, excitation source (21.22 eV), secondary electron cut-off, and relative VBM energies, respectively. **Figure S3**a and b show the UPS spectra in the cut-off and valence regions, respectively, for $MAPbI_3$. Figure S3c and d show the UPS spectra in the cut-off and valence regions, respectively, for CdSe-ZnS-QD (645). Figure S3e and f show the UPS spectra in the cut-off and valence regions, respectively, for the CdSe-ZnS-QD (560). The E_{cutoff} and E_{rel} of the $MAPbI_3$ were estimated to be 16.70 and 1.01 eV, respectively. The E_{cutoff} and E_{rel} of the CdSe-ZnS-QD (645) were estimated to be 18.64 and 3.41 eV, respectively. The E_{cutoff} and E_{rel} of the CdSe-ZnS-QD (560) were 17.90 and 2.79 eV, respectively. Thus, the E_{VBM} of $MAPbI_3$, CdSe-ZnS-QD (645), and CdSe-ZnS-QD (560) were estimated to be approximately −5.53, −5.99, and −6.11 eV, respectively.

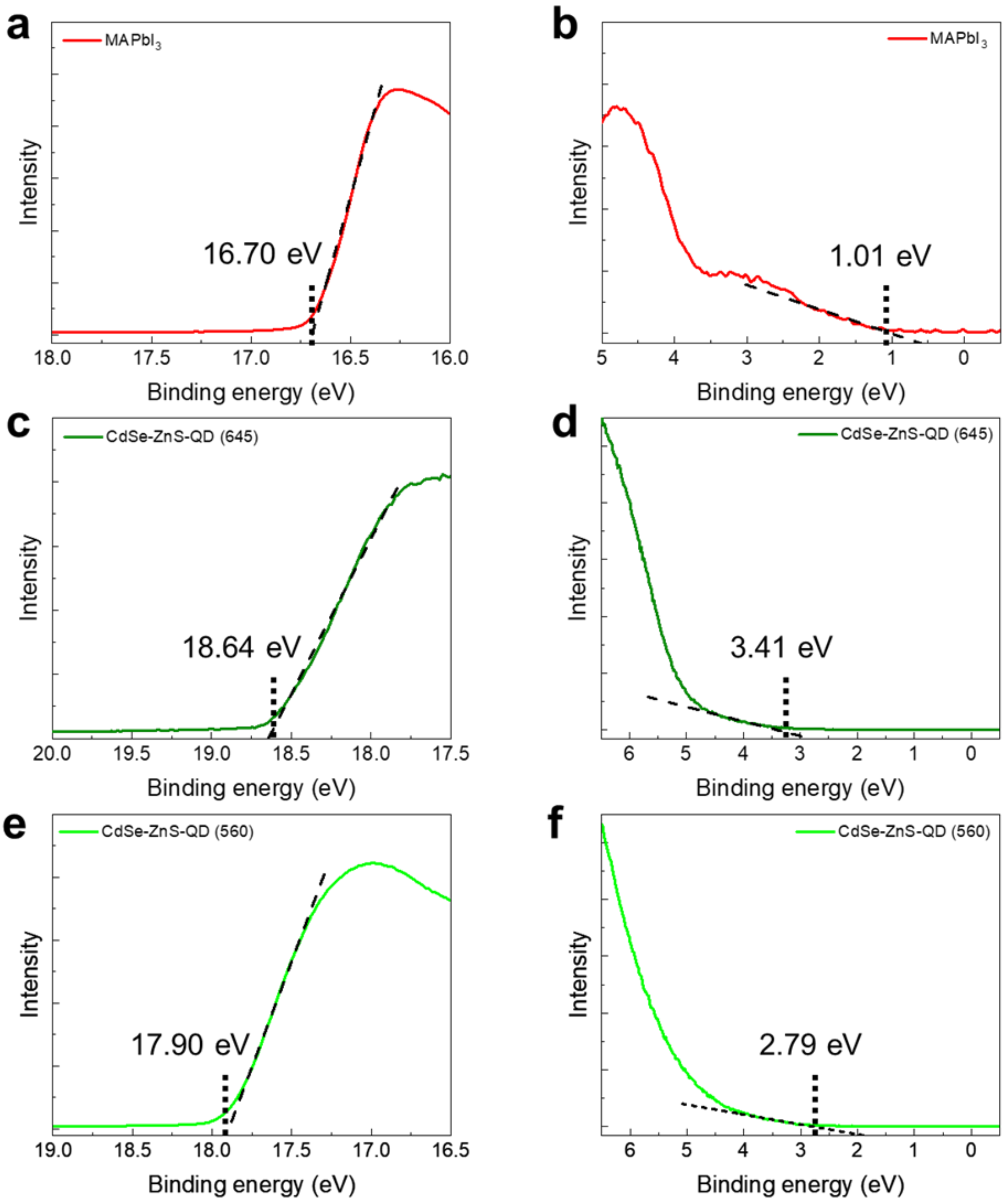

Figure S3. Ultraviolet photoelectron spectra (UPS) for the $MAPbI_3$ in a) cut-off and b) valence regions. UPS spectra of the CdSe-ZnS-QD (645) in c) cut-off and d) valence regions. UPS spectra of the CdSe-ZnS-QD (560) in e) cut-off and f) valence regions.

Section S4. Absorbance and PL spectra for the $MAPbI_3$, CdSe-ZnS-QD (645), and CdSe-ZnS-QD (560)

Figure S4a and b show the absorbance and PL spectra, respectively, of the $MAPbI_3$ (red), CdSe-ZnS-QD (645) (green), and CdSe-ZnS-QD (560) (black) at 297 K. The 488 nm (= 2.54 eV) excitation laser was used to obtain the PL spectra. The energy of the conduction band minimum (CBM) is estimated as follows:

$$E_{CBM} = E_{VBM} + E_g, \qquad \text{(S2)}$$

where E_{CBM} and E_{g} are the CBM energy and energy band gap, respectively. With the absorbance edge of $MAPbI_3$ (1.65 eV), CdSe-ZnS-QD (645) (1.95 eV), and CdSe-ZnS-QD (560) (2.26 eV), the E_{CBM} of $MAPbI_3$, CdSe-ZnS-QD (645), and CdSe-ZnS-QD (560) were evaluated to be approximately −3.88, −4.04, and −3.85 eV, respectively. Therefore, the energy band structure of the type-II (type-I) EBA consisting of $MAPbI_3$ and CdSe-ZnS-QD (645) ($MAPbI_3$ and CdSe-ZnS-QD (560)) was obtained, as shown in Figure 1d (Figure 1e).

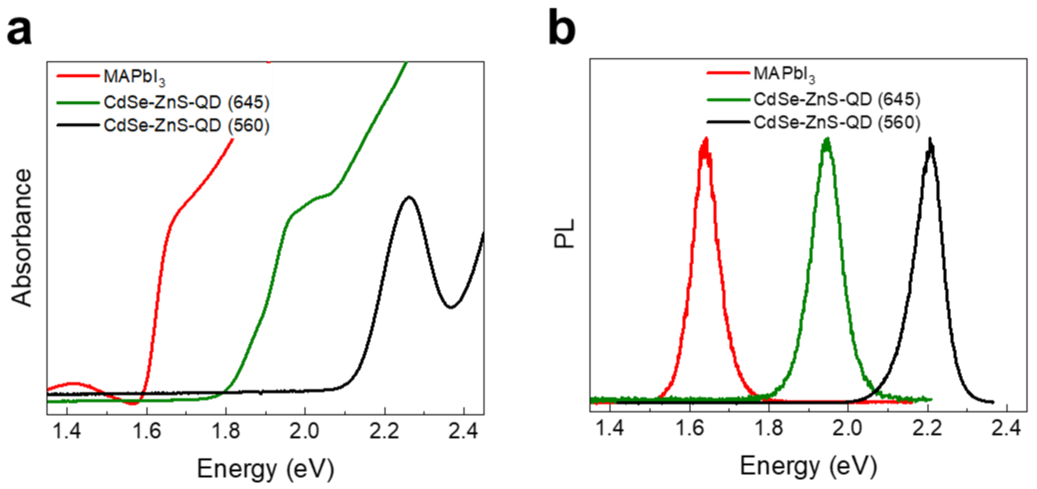

Figure S4. a) Absorbance and b) PL spectra for the $MAPbI_3$ (red curve), CdSe-ZnS-QD (645) (green curve), and CdSe-ZnS-QD (560) (black curve).

Section S5. Peak deconvolution of PL spectrum of $MAPbI_3$ at 3 K

Figure S5 shows the PL spectrum of the $MAPbI_3$ at 3 K. The deconvoluted PL curves with three peaks obtained using Voigt profile fitting were related to the orthorhombic, tetragonal, and defect phases, denoted as $MAPbI_3$-O (magenta curve; 1.64 eV), $MAPbI_3$-T (red curve; 1.58 eV), and $MAPbI_3$-D (orange curve; 1.52 eV), respectively.

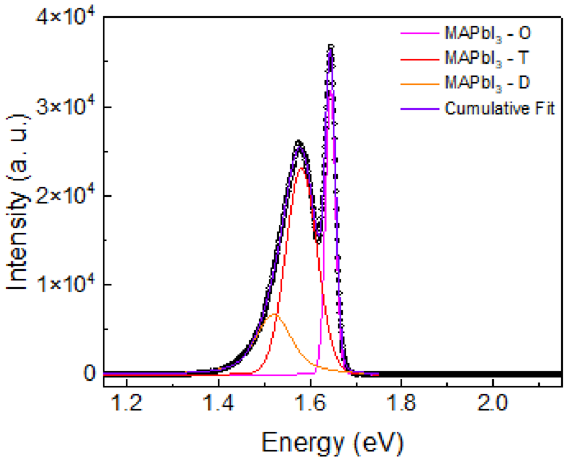

Figure S5. PL spectrum of the $MAPbI_3$ at 3 K with the deconvoluted curves corresponding to orthorhombic (O), tetragonal (T), and defect (D) phases.

Section S6. PL spectra at 50 K

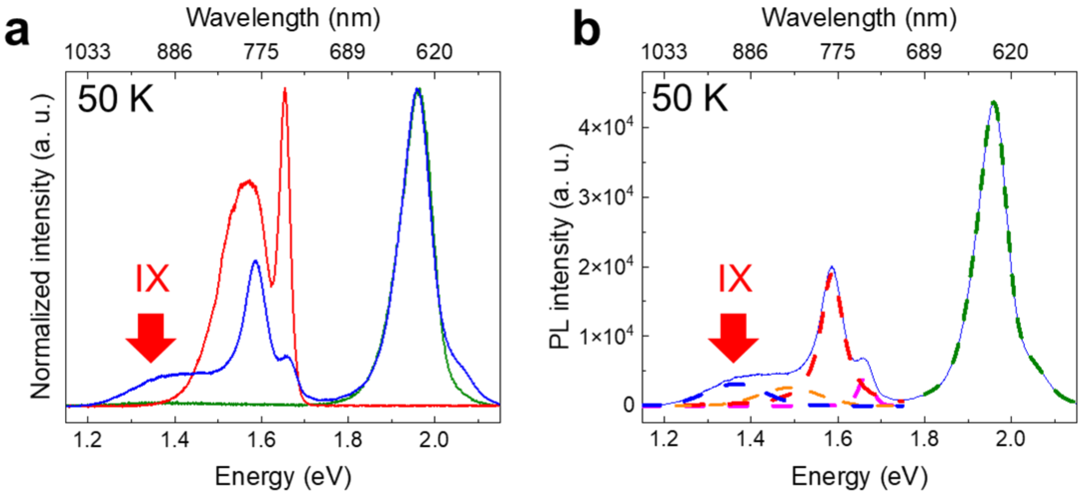

Figure S6. a) PL spectra of the CdSe-ZnS-QD (645) (green curve), $MAPbI_3$ (red curve), and their HS (blue curve) at 50 K. b) PL spectra of the $MAPbI_3$/CdSe-ZnS-QD (645) HS at 50 K with five deconvoluted curves: CdSe-ZnS-QD (645) (green dashed curve; 1.96 eV = 632 nm), $MAPbI_3$-O (magenta dashed curve; 1.66 eV = 747 nm), $MAPbI_3$-T (red dashed curve; 1.59 eV = 780 nm), $MAPbI_3$-D (orange dashed curve; 1.49 eV = 833 nm), and IXs (blue dashed curve; 1.36 eV = 909 nm) at 50 K.

Section S7. Lifetime of intralayer excitons and IXs

Time-resolved PL (tr-PL) decay curves were analyzed using a multi-exponential model given as

$$I(t) = \sum_i A_i \exp(-t/\tau_i), \qquad \text{(S3)}$$

where I, A, and τ denote the intensity, amplitude, and decay time, respectively. The average lifetime (τ_{avg}) was estimated as

$$\tau_{avg} = \sum_i A_i \tau_i / \sum_i A_i. \qquad \text{(S4)}$$

The τ_{avg} values of the intralayer excitons and IXs can be obtained from bi- and single-exponential decay models. The tr-PL spectrum of $MAPbI_3$ was fitted using an incomplete bi-exponential decay model, as follows:[2]

$$I(t) = \frac{A_1}{1-\exp(-t_0/\tau_1)} \exp(-t/\tau_1) + \frac{A_2}{1-\exp(-t_0/\tau_2)} \exp(-t/\tau_2), \qquad \text{(S5)}$$

where t_0 is the repetition time of the pulse laser, set to 50 ns. The parameters obtained from the PL decay curves at 3 K are presented in **Table S1**.

Table S1. Amplitude (A), decay time (τ), and estimated average lifetime (τ_{avg}) of the excitons of CdSe-ZnS-QD (645), $MAPbI_3$ and IXs of HS obtained from Figure 2c and d.

	A_1	τ_1 (ns)	A_2	τ_2 (ns)	τ_{avg} (ns)
CdSe-ZnS-QD (645)	1.02	0.478	0.238	1.73	0.715
$MAPbI_3$	0.126	1.53	0.0464	865	234
$MAPbI_3$/CdSe-ZnS-QD (645) HS	0.979	5680	-	-	5680

Figure S7 shows the tr-PL spectra in μs-scale for $MAPbI_3$ for defect states (red curve) and IXs (blue curve) of the HS. The τ_{avg} of the $MAPbI_3$ with defect states and the IXs of the HS were estimated to be about 2.03 and 5.68 μs, respectively.

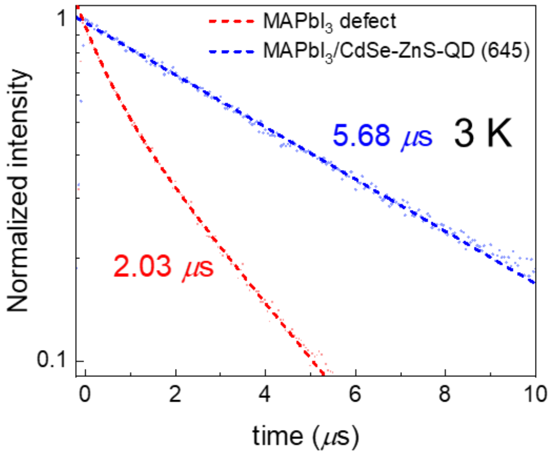

Figure S7. tr-PL spectra of the defect of pristine $MAPbI_3$ (red curve) and IXs of $MAPbI_3$/CdSe-ZnS-QD (645) HS (blue curve) at 3 K.

Section S8. Charge transfer analysis using tr-PL and PL spectra of CdSe-ZnS-QD (645) at 3 K

The charge (hole) transfer rate (k_{HT}) and its efficiency (E_{HT}) from QDs can be estimated as follows:[3,4]

$$k_{HT} = \frac{1}{\tau_{QD_{HS}}} - \frac{1}{\tau_{QD_{pristine}}}, \quad \text{(S6)}$$

$$E_{HT} = 1 - \frac{\tau_{QD_{HS}}}{\tau_{QD_{pristine}}}, \quad \text{(S7)}$$

where τ_{QDHS} is average lifetime of QDs in HS and $\tau_{QDpristine}$ is that of pristine QDs. **Figure S8**a and b show tr-PL and PL spectra at 3 K, respectively, of CdSe-ZnS-QD (645) of the pristine (black) and in HS (red). The k_{HT} and E_{HT} of CdSe-ZnS-QD (645) were estimated to be approximately 9.05 × 10^8 s^{-1} and 64.0% using the τ_{QDHS} = 0.707 ns and $\tau_{QDpristine}$ = 1.96 ns as shown in Figure S8a. In addition, the relative charge transfer efficiency (R_{CT}) using the PL intensity can be estimated using

$$R_{CT} = 1 - \frac{I_{QD_{HS}}}{I_{QD_{pristine}}}, \quad \text{(S8)}$$

where I_{QDHS} and $I_{QDpristine}$ are integrated PL intensities from the QDs in HS and the pristine QDs, respectively. The R_{CT} was estimated to be 68.7%, which is similar value (64.0%) to the E_{HT}.

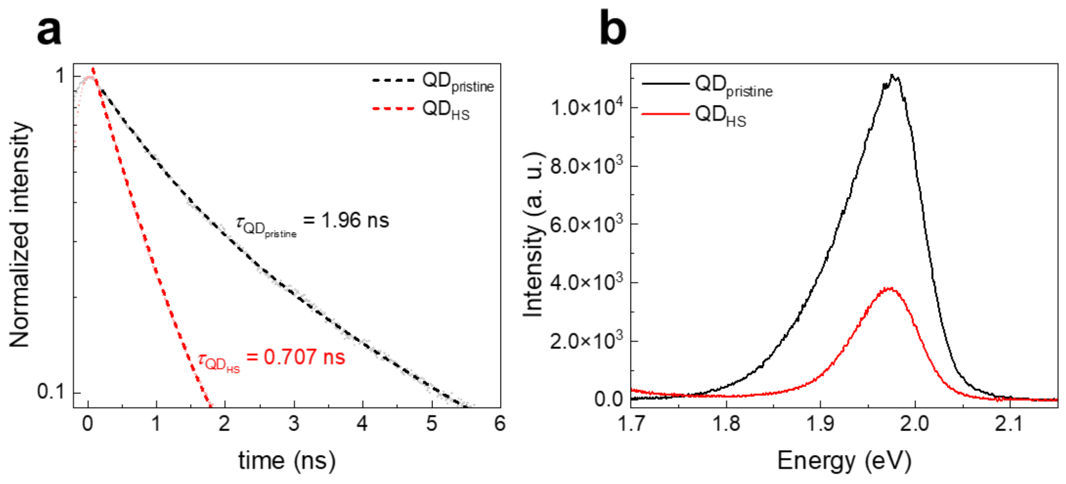

Figure S8. a) tr-PL and b) PL spectra of CdSe-ZnS-QD (645) before (black) and after (red) hybridization with $MAPbI_3$, measured under 3 K.

Section S9. Magnification of PL spectra of IXs at 3 K

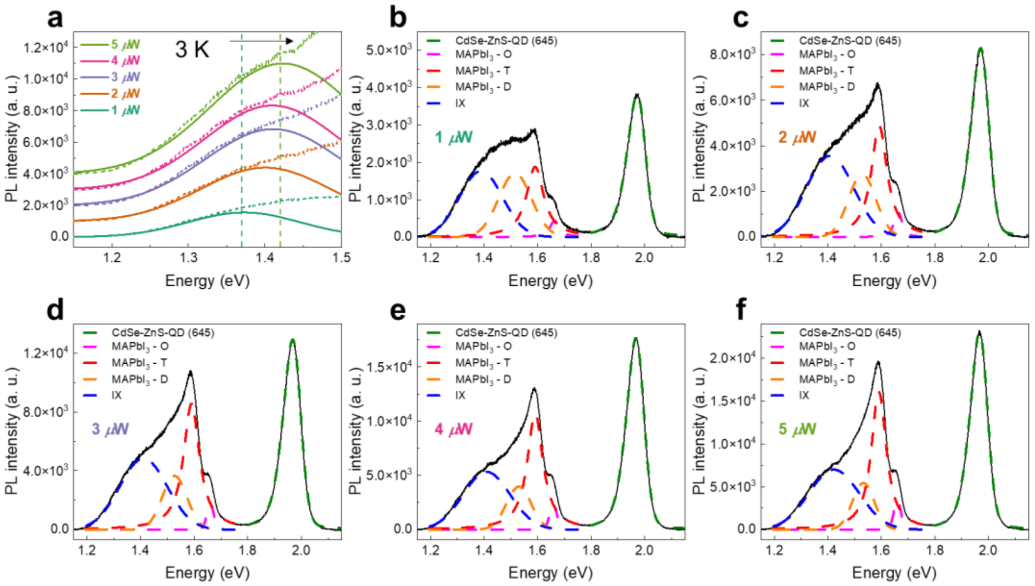

Figure S9. a) Magnification of PL spectra of $MAPbI_3$/CdSe-ZnS-QD (645) HS with various excitation powers from 1 to 5 μW in the range of 1.15 – 1.5 eV focusing on IXs. b) – f) PL spectra of $MAPbI_3$/CdSe-ZnS-QD (645) HS with deconvoluted curves at various excitation powers.

Section S10. Binding energy of IXs

Binding energy of IXs can be estimated using Arrhenius relation given as:[5,6]

$$I(T) = \frac{I_0}{1+\exp(-E_b/k_B T)}, \quad \text{(S9)}$$

where I, T, I_0, E_b, and k_B are the intensity, temperature, intensity at 0 K, binding energy, and Boltzmann constant, respectively. **Figure S10** shows the intensity of the IXs as a function of inverse temperature. The binding energies of the far-red IXs were estimated to be approximately 15.3 meV. According to previous reports, the binding energies of the intralayer excitons of CdSe-ZnS-QD and $MAPbI_3$ were 150 – 200 meV[7,8] and 19 – 50 meV,[9–12] respectively. The binding energies of the IXs were relatively weaker than those of the intralayer excitons.[13]

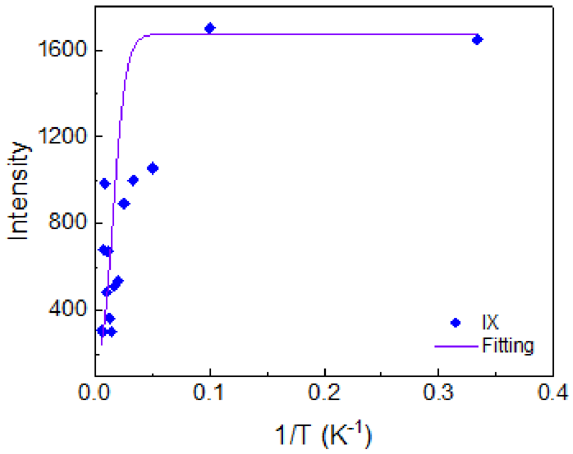

Figure S10. Intensity of IXs of the $MAPbI_3$/CdSe-ZnS-QD (645) HS as a function of inverse temperature based on Arrhenius relation.

Section S11. Temperature dependent PL spectra of $MAPbI_3$

Figure S11a shows the temperature-dependent PL spectra of $MAPbI_3$ from 3 to 180 K. Fluctuation of the PL peak position was observed near 140 K, which is due to the phase transition from the orthorhombic to tetragonal phases of the pristine $MAPbI_3$.[14–16] Figure S11b shows the PL peak energy of $MAPbI_3$ as a function of temperature.

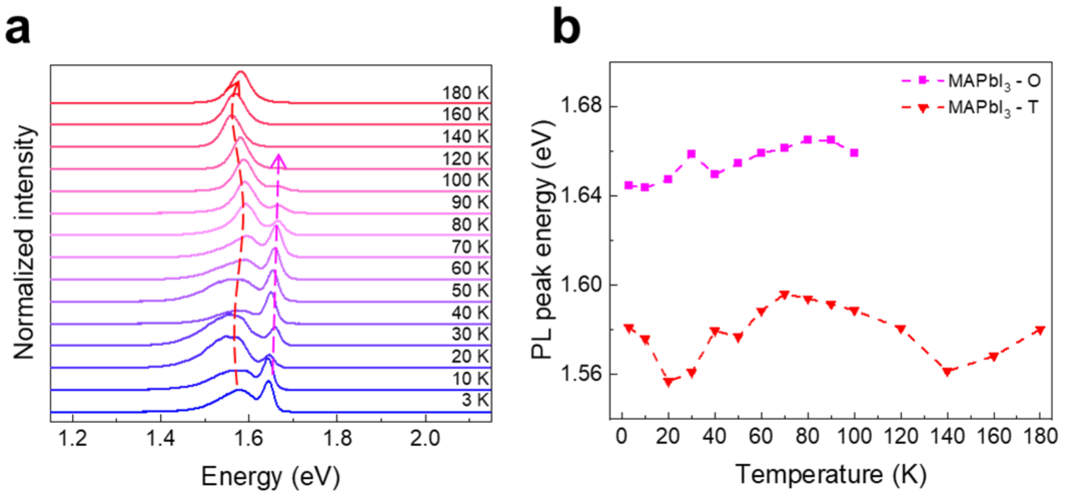

Figure S11. a) PL spectra of the $MAPbI_3$ at various temperatures (3 to 180 K). The arrows are eye guidance for peak shift of $MAPbI_3$-O (magenta) and $MAPbI_3$-T (red). b) PL peak energy as a function of temperature for the $MAPbI_3$-O (magenta) and $MAPbI_3$-T (red).

Section S12. XPS spectra of $MAPbI_3$ and $MAPbI_3$/CdSe-ZnS-QD (645) HS

Figure S12 shows the X-ray photoelectron spectra (XPS) for Pb $4f_{5/2}$ of $MAPbI_3$ (black curve) and $MAPbI_3$/CdSe-ZnS-QD (645) HS (red curve). After hybridization with CdSe-ZnS-QDs, the binding energy of Pb $4f_{5/2}$ was negatively shifted by approximately 100 meV. The negative shift in the binding energy after the formation of HS indicates that the potential applied to the core-level electrons became weaker. In this case, the under-coordinated Pb^{2+} ions were negatively charged owing to the reduction in the halide vacancies. Therefore, the CdSe-ZnS-QD (645) was attributed to $MAPbI_3$ as a passivating layer.

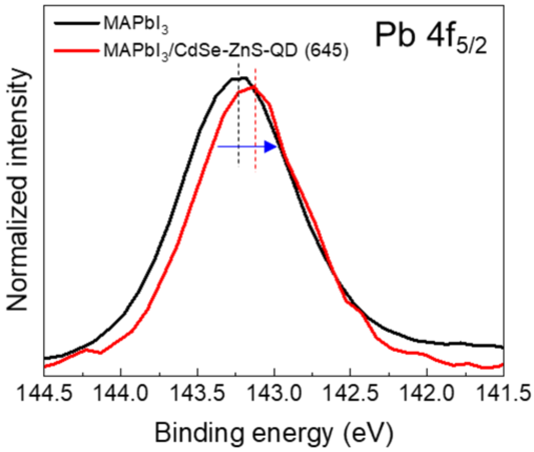

Figure S12. X-ray photoelectron spectra (XPS) for Pb $4f_{5/2}$ of the $MAPbI_3$ before (black curve) and after (red curve) hybridization with CdSe-ZnS-QD (645).

Section S13. PL spectra related to phase transition of $MAPbI_3$

The PL peak related to the orthorhombic phase of $MAPbI_3$ was typically observed at 1.65 – 1.7 eV at low temperatures.[15,17,18] As shown in **Figure S13**a, the PL peak corresponding to the $MAPbI_3$-O phase was observed near 120 – 140 K. Thus, the phase transition temperature (T_c) can be assigned as approximately 140 K for $MAPbI_3$. Similarly, the phase transition of $MAPbI_3$/CdSe-ZnS-QD (645) HSs was observed at approximately 90 K, as shown in Figure S13b.

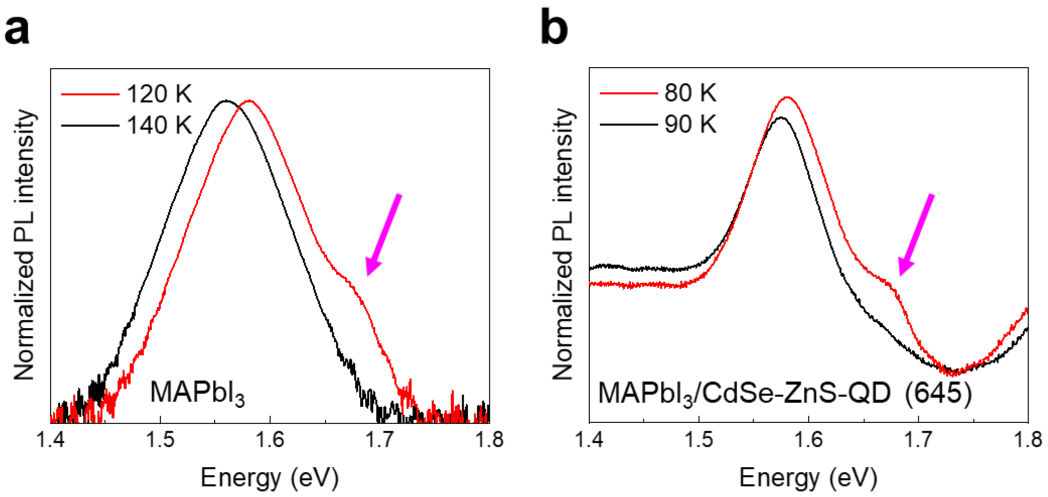

Figure S13. PL spectra at near phase transition temperature (T_c) of the $MAPbI_3$ a) before and b) after the hybridization with CdSe-ZnS-QD (645). The magenta arrows indicate the $MAPbI_3$-O related PL signal.

Section S14. Characteristics of energy gap (E_g)

Figure S14a shows the variation in the energy gap (E_g) with temperature ($\Delta E_g/\Delta T$), demonstrating the relationship between the energy of the IXs and the energy band edges of the constituent layers. The $\Delta E_g/\Delta T$ of the CdSe-ZnS-QD (645) (green) was stable in the measured temperature range, whereas those of $MAPbI_3$ (red) and HS (blue) fluctuated near the phase transition temperature (T_c = 90 K) of $MAPbI_3$ as shown in Figure S14a. The $\Delta E_g/\Delta T$ of $MAPbI_3$ near T_c shows relatively large negative values, which is in accordance with previous reports.[19–22] The PL peak shift ($\Delta E_p = E_{5\,\mu W} - E_{1\,\mu W}$) of the IXs varies with excitation power from 1 to 5 μW at each temperature, as shown in Figure S14b. ΔE_p drastically increased below 90 K, suggesting an increase in the concentration of IXs.[23–26] This implies that the effective Bohr radius of the IXs is relatively larger than that of the intralayer excitons at a specific excitation power below 90 K.

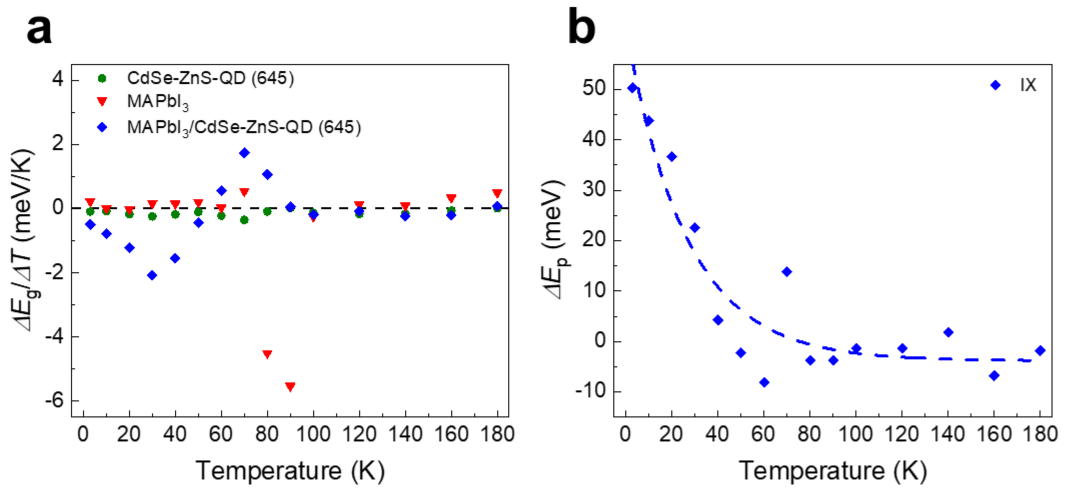

Figure S14. a) $\Delta E_g/\Delta T$ of the CdSe-ZnS-QD (645) (green marker), $MAPbI_3$ (red marker), and $MAPbI_3$/CdSe-ZnS-QD (645) HS (blue marker). b) PL peak shift ($\Delta E_p \equiv E_{5\,\mu W} - E_{1\,\mu W}$) of the IXs varying with excitation power from 1 to 5 μW at below 180 K.

Section S15. *I-V* characteristic curves of pristine $MAPbI_3$ and $MAPbI_3$/CdSe-ZnS-QD (645) heterostructure photodetectors

Figure S15a and b show the *I-V* characteristic curves of pristine $MAPbI_3$ and $MAPbI_3$/CdSe-ZnS-QD (645) HS photodetectors, respectively, at different excitation wavelengths.

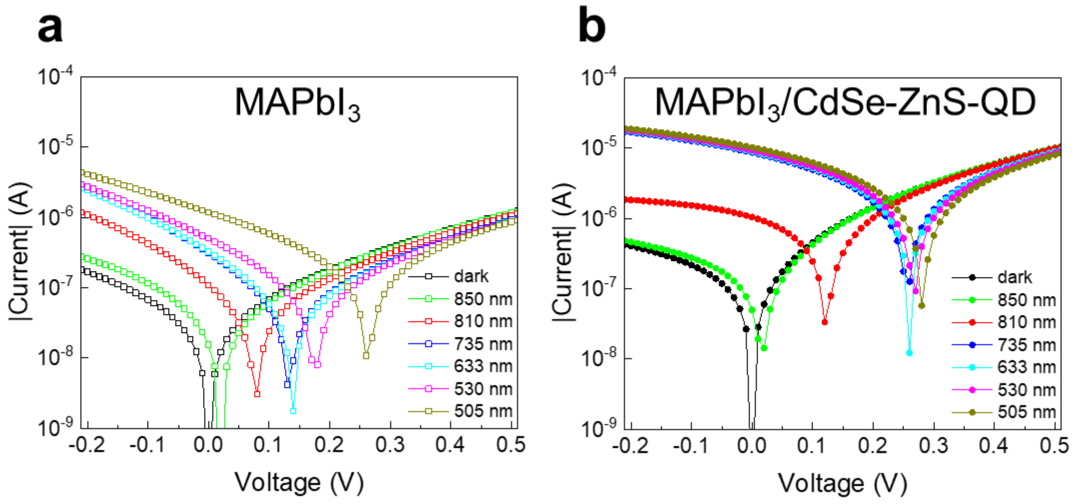

Figure S15. *I-V* characteristic curves of a) pristine $MAPbI_3$ and b) $MAPbI_3$/CdSe-ZnS-QD (645) HS devices at different wavelengths of incident light-irradiation. Black, green, red, blue, cyan, magenta, and brown curves represent the conditions of dark and that of being irradiated with λ_{ex} = 850, 810, 735, 633, 530, and 505 nm, respectively.

Section S16. PL spectra of different batch of $MAPbI_3$/CdSe-ZnS-QD (645) HS

Figure S16a and b show the normalized PL spectra of the different batch of $MAPbI_3$/CdSe-ZnS-QD (645) HS at various temperatures. Temperature dependency of PL spectra including the peak shift in Figure S16 is similar to those of Figure 3e, confirming the results.

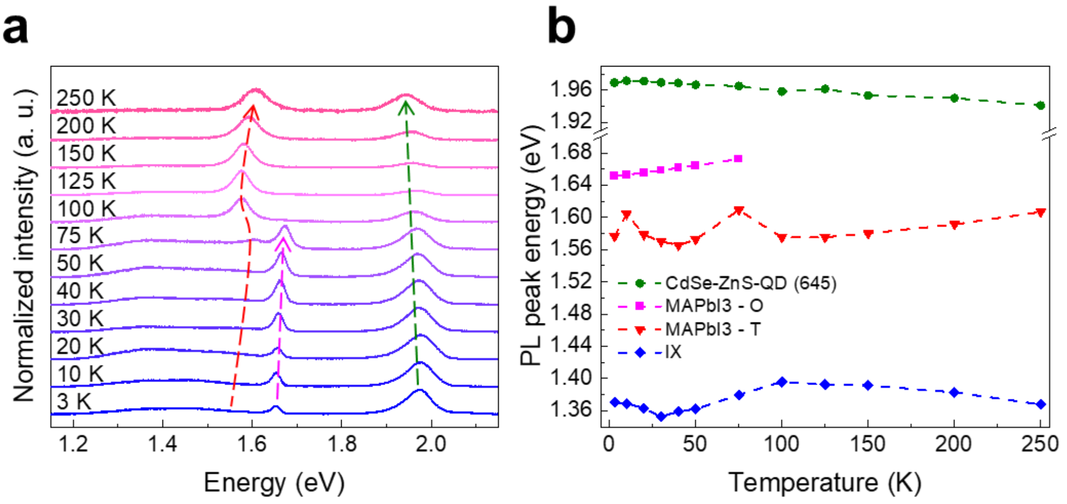

Figure S16. a) Normalized PL spectra of the $MAPbI_3$/CdSe-ZnS-QD (645) HS (different batch) at various temperatures from 3 to 250 K in the range of 1.15 – 2.15 eV. The arrows are eye guidance for peak shift of CdSe-ZnS-QD (645) (green), $MAPbI_3$-O (magenta), and $MAPbI_3$-T (red). b) PL peak position as a function of temperature for the CdSe-ZnS-QD (645) (green), $MAPbI_3$-O (magenta), $MAPbI_3$-T (red), and IXs (blue).

A shift of the IXs PL emission was observed near 80 – 100 K as shown in **Figure S17**.

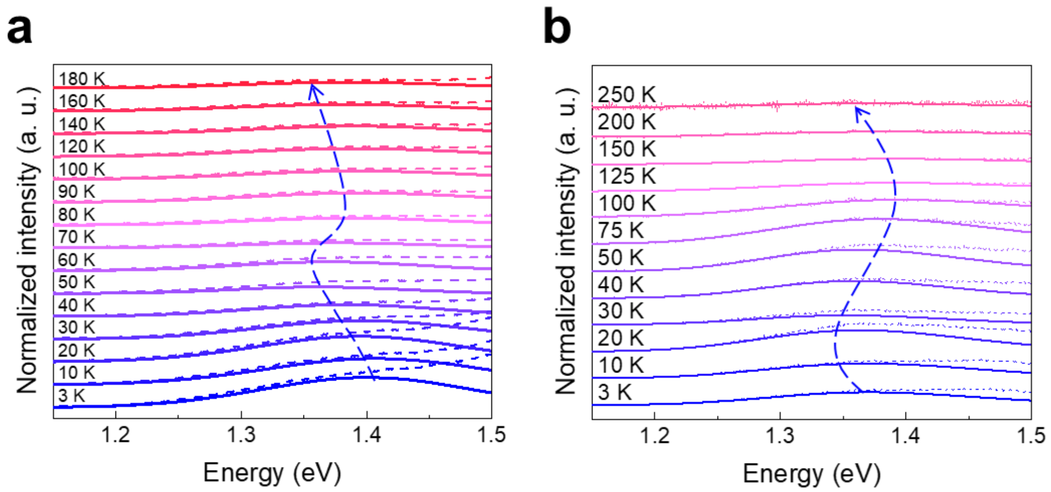

Figure S17. Normalized PL spectra of the $MAPbI_3$/CdSe-ZnS-QD (645) HS at various temperatures in the range of 1.15 – 1.5 eV for the same sample in a) Figure 3d and b) Figure S16a. The arrows are eye guidance for peak shift of IXs (blue).

References

[1] L. Brus, *J. Phys. Chem.* **1986**, *90*, 2555.

[2] R. W. K. Leung, S.-C. A. Yeh, Q. Fang, *Biomed. Opt. Express* **2011**, *2*, 2517.

[3] Z. Xu, C. R. Hine, M. M. Maye, Q. Meng, M. Cotlet, *ACS Nano* **2012**, *6*, 4984.

[4] J.-S. Chen, M. Li, M. Cotlet, *ACS Omega* **2019**, *4*, 9102.

[5] J. Y. Kim, T. J. Kim, S. Lee, E. Lee, J. Kim, J. Joo, *ACS Appl. Nano Mater.* **2022**, *5*, 11167.

[6] J. Park, J. W. Choi, W. Kim, R. Lee, H. C. Woo, J. Shin, H. Kim, Y. J. Son, J. Y. Jo, H. Lee, S. Kwon, C. L. Lee, G. Y. Jung, *RSC Adv.* **2019**, *9*, 14868.

[7] J. M. Elward, A. Chakraborty, *J. Chem. Theory Comput.* **2013**, *9*, 4351.

[8] J. M. Elward, A. Chakraborty, *J. Chem. Theory Comput.* **2015**, *11*, 462.

[9] M. Hirasawa, T. Ishihara, T. Goto, K. Uchida, N. Miura, *Phys. B Condens. Matter* **1994**, *201*, 427.

[10] K. Tanaka, T. Takahashi, T. Ban, T. Kondo, K. Uchida, N. Miura, *Solid State Commun.* **2003**, *127*, 619.

[11] M. Hamada, S. Rana, E. Jokar, K. Awasthi, E. W.-G. Diau, N. Ohta, *ACS Appl. Energy Mater.* **2020**, *3*, 11830.

[12] D. M. Niedzwiedzki, H. Zhou, P. Biswas, *J. Phys. Chem. C* **2022**, *126*, 1046.

[13] P. Rivera, J. R. Schaibley, A. M. Jones, J. S. Ross, S. Wu, G. Aivazian, P. Klement, K. Seyler, G. Clark, N. J. Ghimire, J. Yan, D. G. Mandrus, W. Yao, X. Xu, *Nat. Commun.* **2015**, *6*, 6242.

[14] W. Kong, Z. Ye, Z. Qi, B. Zhang, M. Wang, A. Rahimi-Iman, H. Wu, *Phys. Chem. Chem. Phys.* **2015**, *17*, 16405.

[15] N. Shrestha, Z. Song, C. Chen, E. Bastola, X. Wang, Y. Yan, R. J. Ellingson, *J. Phys. Chem. Lett.* **2020**, *11*, 121.

[16] A. Dobrovolsky, A. Merdasa, E. L. Unger, A. Yartsev, I. G. Scheblykin, *Nat. Commun.* **2017**, *8*, 34.

[17] Y. Liu, H. Lu, J. Niu, H. Zhang, S. Lou, C. Gao, Y. Zhan, X. Zhang, Q. Jin, L. Zheng, *AIP Adv.* **2018**, *8*, 095108.

[18] L. Q. Phuong, Y. Yamada, M. Nagai, N. Maruyama, A. Wakamiya, Y. Kanemitsu, *J. Phys. Chem. Lett.* **2016**, *7*, 2316.

[19] S. Khatun, A. Maiti, G. Das, A. J. Pal, *J. Phys. Chem. C* **2020**, *124*, 19841.

[20] S. Singh, C. Li, F. Panzer, K. L. Narasimhan, A. Graeser, T. P. Gujar, A. Köhler, M. Thelakkat, S. Huettner, D. Kabra, *J. Phys. Chem. Lett.* **2016**, *7*, 3014.

[21] B. Yang, W. Ming, M. Du, J. K. Keum, A. A. Puretzky, C. M. Rouleau, J. Huang, D. B. Geohegan, X. Wang, K. Xiao, *Adv. Mater.* **2018**, *30*, 1705801.
[22] W. Li, J. Tang, D. Casanova, O. V. Prezhdo, *ACS Energy Lett.* **2018**, *3*, 2713.
[23] L. A. Jauregui, A. Y. Joe, K. Pistunova, D. S. Wild, A. A. High, Y. Zhou, G. Scuri, K. De Greve, A. Sushko, C.-H. Yu, T. Taniguchi, K. Watanabe, D. J. Needleman, M. D. Lukin, H. Park, P. Kim, *Science.* **2019**, *366*, 870.
[24] P. Nagler, G. Plechinger, M. V. Ballottin, A. Mitioglu, S. Meier, N. Paradiso, C. Strunk, A. Chernikov, P. C. M. Christianen, C. Schüller, T. Korn, *2D Mater.* **2017**, *4*, 025112.
[25] W. Li, X. Lu, S. Dubey, L. Devenica, A. Srivastava, *Nat. Mater.* **2020**, *19*, 624.
[26] B. Laikhtman, R. Rapaport, *Phys. Rev. B* **2009**, *80*, 195313.
